# FAIR compliant database development for human microbiome data samples

**DOI:** 10.3389/fcimb.2024.1384809

**Published:** 2024-05-07

**Authors:** Mathieu Dorst, Nathan Zeevenhooven, Rory Wilding, Daniel Mende, Bernd W. Brandt, Egija Zaura, Alfons Hoekstra, Vivek M. Sheraton

**Affiliations:** ^1^ Informatics Institute, University of Amsterdam, Amsterdam, Netherlands; ^2^ Supabase Limited Liability Company (LLC), San Francisco, CA, United States; ^3^ Amsterdam Institute of Infection and Immunity, Amsterdam University Medical Center, Amsterdam, Netherlands; ^4^ Department of Preventive Dentistry, Academic Centre for Dentistry Amsterdam, Vrije Universiteit Amsterdam and University of Amsterdam, Amsterdam, Netherlands; ^5^ Computational Science Lab, Informatics Institute, University of Amsterdam, Amsterdam, Netherlands

**Keywords:** database, fair principles, general data protection regulation (GDPR), (meta)data, microbiome, pseudonymize, real-time

## Abstract

**Introduction:**

Sharing microbiome data among researchers fosters new innovations and reduces cost for research. Practically, this means that the (meta)data will have to be standardized, transparent and readily available for researchers. The microbiome data and associated metadata will then be described with regards to composition and origin, in order to maximize the possibilities for application in various contexts of research. Here, we propose a set of tools and protocols to develop a real-time FAIR (Findable. Accessible, Interoperable and Reusable) compliant database for the handling and storage of human microbiome and host-associated data.

**Methods:**

The conflicts arising from privacy laws with respect to metadata, possible human genome sequences in the metagenome shotgun data and FAIR implementations are discussed. Alternate pathways for achieving compliance in such conflicts are analyzed. Sample traceable and sensitive microbiome data, such as DNA sequences or geolocalized metadata are identified, and the role of the GDPR (General Data Protection Regulation) data regulations are considered. For the construction of the database, procedures have been realized to make data FAIR compliant, while preserving privacy of the participants providing the data.

**Results and discussion:**

An open-source development platform, Supabase, was used to implement the microbiome database. Researchers can deploy this real-time database to access, upload, download and interact with human microbiome data in a FAIR complaint manner. In addition, a large language model (LLM) powered by ChatGPT is developed and deployed to enable knowledge dissemination and non-expert usage of the database.

## Graphical Abstract

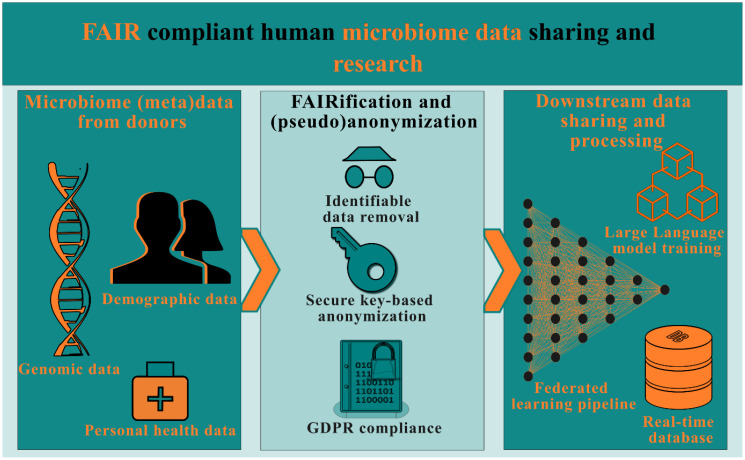

## 1 Introduction

### 1.1 Data and data sharing

Data sharing facilitates collaboration between researchers and therefore could lead to new findings as various disciplines and research groups utilize the data differently (Yoong et al., 2022). The FAIR principles are standards by which data can be more easily exchanged, preserved, and curated for research purposes (Chue Hong et al., 2021). The downstream reuse of data would often run into significant issues, such as incomplete metadata, absence of raw data, or incompatibility of software, leading to datasets being practically unusable outside of the primary research case for which they were conceived (Roche et al., 2015). Crucially, in development of machine learning techniques, the efficiency of the algorithms relies heavily on the quality of the labels (Willemink et al., 2020; Wilding et al., 2022). In such cases, standardizing the metadata would enable the generation of high quality labels and help systematize the label generation process. Machines can then more easily query the database for the right data and perform operations on it, while researchers, for example, can easily combine datasets to get new insights. This removes the friction that exists with different data formats and gives space for more efficient and faster data handling, which results in new insights to evolve more rapidly (Abuimara et al., 2022). Large numerical datasets, such as data originating from multiscale simulation studies (Sheraton et al., 2019; Béquignon et al., 2023), -omics (Subramanian et al., 2020) or imaging studies (Bray et al., 2017; Baglamis et al., 2023), should be properly categorized along with clearly described provenance. Additionally, the origin and composition of the data will have to be described, to enable data reconstructions in such a way that it cannot be misinterpreted. To overcome these obstacles of non-standardized or flawed use of data and to ensure proper data sharing, the Findability, Accessibility, Interoperability and Reusability (FAIR) principles were introduced (Wilkinson et al., 2016). A FAIR compliant database makes it very easy for all types of data to be discovered, since the metadata has been standardized (Da Silva Santos et al., 2023). Every FAIR object (image, table, text, etc.) or dataset should have a unique identifier assigned, which should then be described with rich metadata. In a study evaluating the FAIR4HEALTH initiative, the implementation of FAIR principles has been proven to save researchers on average approximately 56% of their time in data gathering and compilation activities and approximately 16,800 euros per month in institution funding, when conducting health research efforts (Martínez-García et al., 2023). There are additional contextual advantages such as avoidance of the risk of repetition of research and expedited literature gathering (Garabedian et al., 2022). FAIR compliance or open-data availability requirements for scientific research is gradually becoming crucial. There are requirements set out by funding agencies and journals to make data open source. Advanced planning for FAIR set-up is crucial to keep costs and implementation durations at minimal. Retrospective FAIRification processes, such as in pharmaceutical research and development (R&D) departments, have been show to entail significant costs (Alharbi et al., 2021). Assuming a 2.5% cost of total project budget for FAIR implementation, it has been shown to save around €2.6 billion per year for EU Horizon 2020 projects (European Commission, 2018). It is therefore imperative to incentivize and initiate FAIR deployment at the early stages of a project rather than towards the end of life of a project or after publication of research.

### 1.2 FAIR principles in the context of human microbiome data

Here, the focus is on application of the FAIR principles to human microbiome research. “Microbiome data” refers to the genetic material that is collected and analyzed from a community of microorganisms, such as bacteria, viruses, fungi, and other microbes, that live in a specific environment. Techniques such as microbiome shotgun sequencing is one of the methods to generate microbiome data. This involves breaking down the genetic material from all the microorganisms in a sample into small fragments, or reads. By comparing these reads to reference databases, researchers can identify and quantify the different microorganisms present in the sample, as well as determine the functions of the genes that are present. When looking at microbiome data, for which, in this case, the microbiome will be defined according to Berg et al. (2020) as “all of the microbial components in a given ecosystem or plant, animal or human system”, a few issues arise with FAIR compliance. There are microbiome databases such as National Microbiome Data Collaborative (NMDC) (The National Microbiome Data Collaborative Data Portal: an integrated multi-omics microbiome data resource, 2022) that focus on providing a FAIR-adherent platform for storage of microbiome meta data. Microbiome data from non-human systems, such as plant-associated or aquatic ecosystems, which are not bound by intellectual property (IP) and ethical considerations, could be readily uploaded to these platforms with minimal filtering or data clean-up. However, the microbiome (meta)data derived from humans, contains highly confidential information such as fragments of human DNA. Consequently, it is not permissible to disclose this data to the general public (Irving and Clarke, 2019). If this pseudonymized or anonymized data were to be made public and combined with the metadata of the dataset, which includes information regarding for example the age, sex and (approximate) location of the donor, it could lead to an intrusion on the privacy of the donor, thus violating the GDPR (Gürsoy et al., 2022). To enable open data publications, human (host) DNA data scrubbing tools have been proposed. However, such tools do not remove all human host DNA. Additionally, during this process, the tools may incorrectly remove some non-host DNA data. Bad actors could potentially use this partly scrubbed datasets and their derivatives to orchestrate serious privacy violations. For example, the identity of the donor could be derivable, and private medical information could be traceable, such as possible health risks and genetic mutations (Irving and Clarke, 2019).

**Figure 1:**
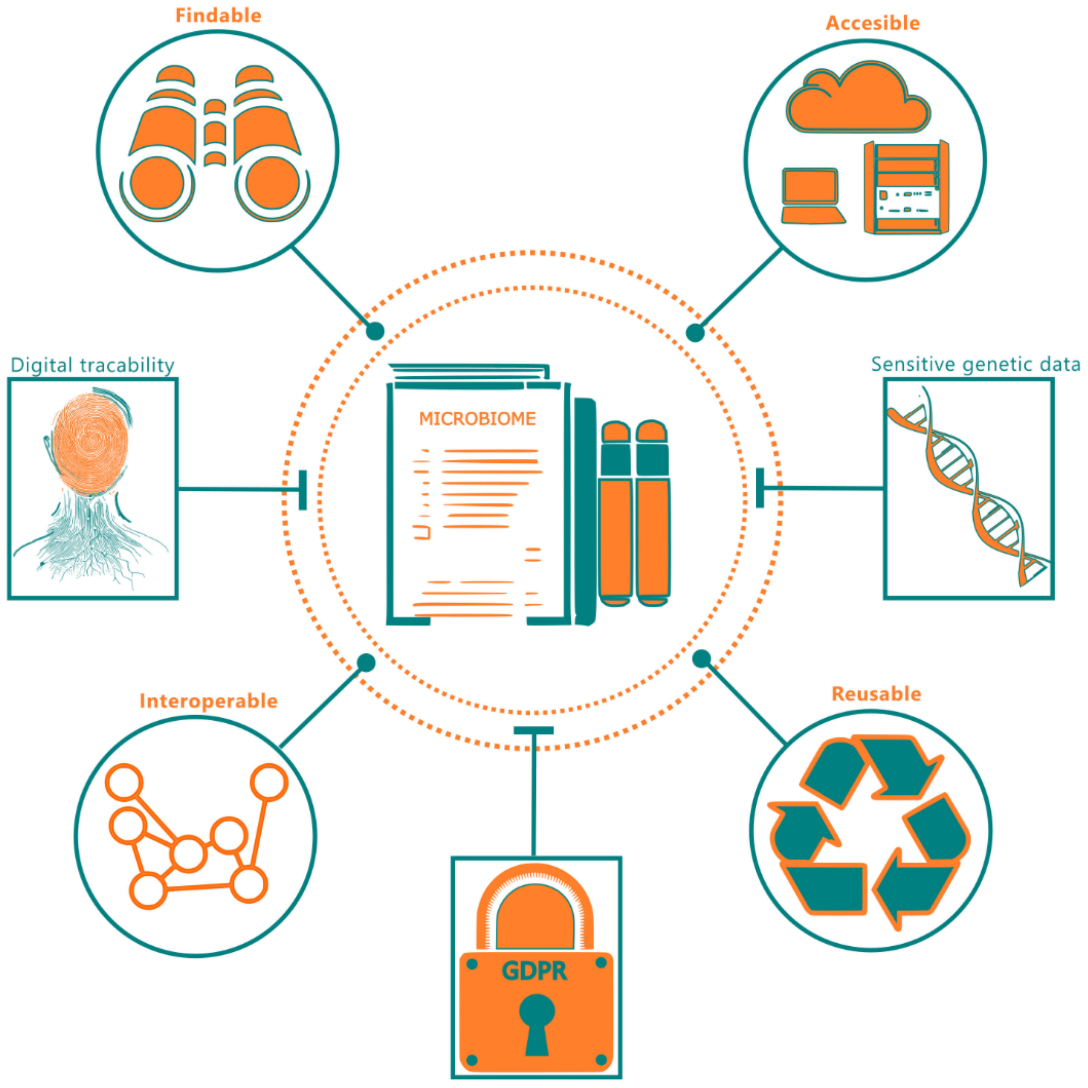
A summary of FAIR principles with respect to microbiome data. The end-dotted connections to microbiome data indicate the FAIR requirements and the flathead connections indicate the practical considerations inhibiting FAIR deployment.

Development of a FAIR database for human microbiome data begins with establishment of unique identifiers. An object’s unique identifier in a microbiome dataset could be a pseudonymized identifier of a participant or a sample collection location, so that longitudinal data can be traced in the metadata fields. These unique identifiers are essential and can therefore not be redacted. **Figure 1** summarizes the FAIR requirements for microbiome data and practical difficulties associated with implementing such a system with complete data transparency. A general question when comparing the suggestions for data transparency, as proposed by the FAIR principles, and the current GDPR privacy and data legislation, is whether the two can coexist in a world where reuse and transparency of scientific data is fully optimized, and the privacy of donors is guaranteed. This means that the GDPR must be satisfied prior to implementing FAIR principles. Here, we report on development and use of computational and data management tools for striking a balance on implementing the various FAIR principles without violating the privacy regulations.

## 2 Methods and Protocols

This section presents the protocols and tools we have designed for use in the development of FAIR-complaint database for human microbiome data. The protocols, codes and algorithms are available through GitHub at https://github.com/SheratonMV/FAIRDatabase.

### 2.1 Database development

To successfully apply the FAIR principles to a database, the type of database used is crucial. Microbiome data collection and storage is a continuous process. This necessitates the database to be real-time, so the data could be synchronized at all times. Any new additions or changes done at sample collection level or processing level would therefore immediately be reflected in the database, thus ensuring accurate data for all users at all times. Next, to be FAIR-compliant the database system itself should be open source. We rely on Supabase, an open-source, real-time, relational database, which suits our microbiome data well.

Since we handle sensitive (identifiable) information, the database was deployed locally as to handle data in accordance with GDPR guidelines. Additionally, we created a user interface to access the database and upload new data. The database is built with Python programming language (v3.10) and the corresponding supabase_py (v0.02) module (Supabase Inc,). Supabase offers different authentication options for database access. For our microbiome database, users can register with an email address and password and later login with same credentials and additional security measures such as two factor authorization or Single Sign-On (SSO). The login and registration are handled by Supabase functions, which are linked to dedicated authentication for the tables within the database. After signing up, the user does not get access to all functionalities (e.g. uploading is not possible for a standard user, and data collected by an uploader may not be available for other uploaders), and access rights can be modified by the database moderator. Such restriction ensures that raw data entry is carried out only at the sample collection point and prohibits data corruption or manipulation by an intermediate user or the uploader themself.

Unfortunately, Supabase currently neither offers table creation outside of their own SQL-editor nor direct SQL querying. To enable users to upload data in the database, the Python script for the user interface had to be linked to a local SQL editor, which can communicate with the database via SQL scripts directly. For this connection, the open source Psycopg2 (version 2.9.6) package was used (Varrazzo,). This package offers direct connection to the database and can execute SQL queries. The file uploaded is checked for the right format, and then stored into the database. If the format does not comply with FAIR principles, the file is first converted to complaint formats for data and metadata. Currently, the table creation is limited by a soft lock on the Supabase database, to1664 columns per table. In our database, 500 columns will be set as a limit to an uploaded table. The remaining available columns could later be used to add foreign keys to establish relationship between various tables. If the table consists of more columns, the table will be split up in multiple tables. The uploaded tables can be previewed via the user interface. This preview shows the first 15 rows and 10 columns to give the user a visual idea of what the table contains, as well as an overview of the metadata of the table contents. The entire table or associated datasets can be downloaded in CSV format, in accordance with the accessibility principle. To find, filter and carry out privacy metric calculations on the data, based on fields (column names) or metadata, the user can also query the database on certain columns (i.e. DNA sequences) and find corresponding datasets with the specified (pseudo- or anonymized) data.

### 2.2 Data pipeline

**Figure 2:**
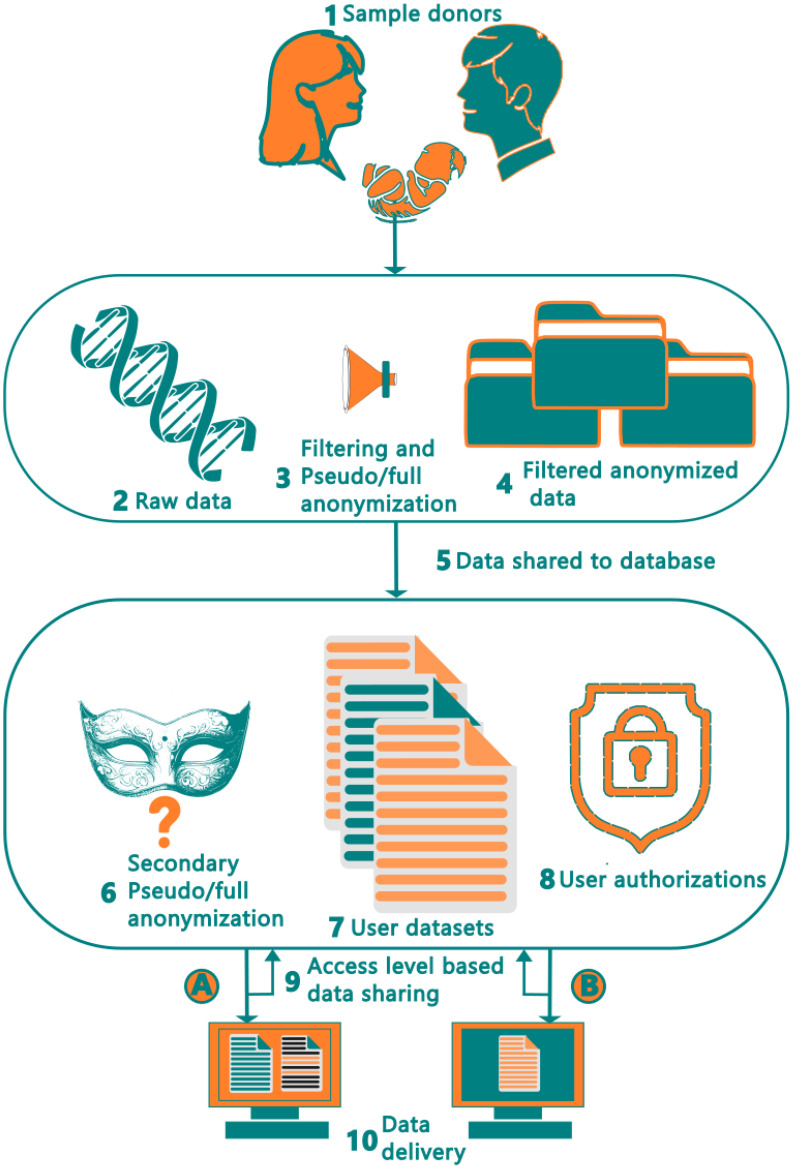
Human microbiome data collection workflow and data transfer pipeline from the donors to end users. The raw data at step 2 includes human shotgun data, metadata and host-associated data such as age, ethnicity, health status.

**Figure 2** shows the complete ten-step data pipeline, starting from the sample collection from the donors and ending at the end-user. The donor dataset containing microbiome data (metagenomics shotgun data) is first filtered to remove all identifiable human (DNA) data using host contamination removal tool such as HoCoRT (Rumbavicius et al., 2023). The samples are then pseudo- or anonymized, depending on the data handling requirements, followed by removal of non-unique and low-quality reads (Rumbavicius et al., 2023). This process is described in **Figure 2** at Step 3. Once this is complete, the data is imported from the environment of the provider (shown as the top rounded square) into our system (shown as the bottom rounded square). Upon entering our environment, and prior to data insertion into the database, a second round of quality control is performed. Here, as seen in Step 6, the data is filtered, with the aim of preventing any sensitive reads accidentally entering our database including checks with the data privacy module. It is also pseudo- or anonymized for a second time, so that, in the case of a leak at our provider, there is no connection to our version of the data, or vice versa. Step 6 is crucial to guaranteeing compliance with GDPR data regulations. Once this step has been completed, the data is inserted into the database, where it is stored and integrated with existing data entries, based on the unique subject identifier, which will be used as the primary key (step 7).

Once the data has been entered into the database, it can be queried by the database users, subject to permissions received based on their authorization level. Once signed in, a user can query and view data, as shown in Step 8 and 9 of **Figure 2**. Users have varying abilities to retrieve and view data based on their authorization levels, with specific examples being demonstrated in steps 9 and 10 using authorization levels A and B respectively. Thus, in the developed pipeline, microbiome data could be anonymized at three stages, 1. Filtering and removing donor’s personal data (association) from the sequencing data with standardized keys at data provider site 2. Removal of human DNA data from metagenomics shotgun data using host contamination removal tool. And 3. Secondary anonymization or pseudonymization at the FAIR database environment (site).

### 2.3 Large language model deployment

Utilizing Supabase’s inbuilt postgres vector database and AI toolkit (Supabase Inc,), we developed an interface to interact with the database relying on a large language model (LLM). The current implementation relies on scaffolding an edge function to forward query to ChatGPT’s API access. The LLM vendor or software can be switched by changing the API. Thus, the LLM could be locally hosted and queried from the database, in the future, provided sufficient computing resources are available.

### 2.4 Throughput results

**Figure 3:**
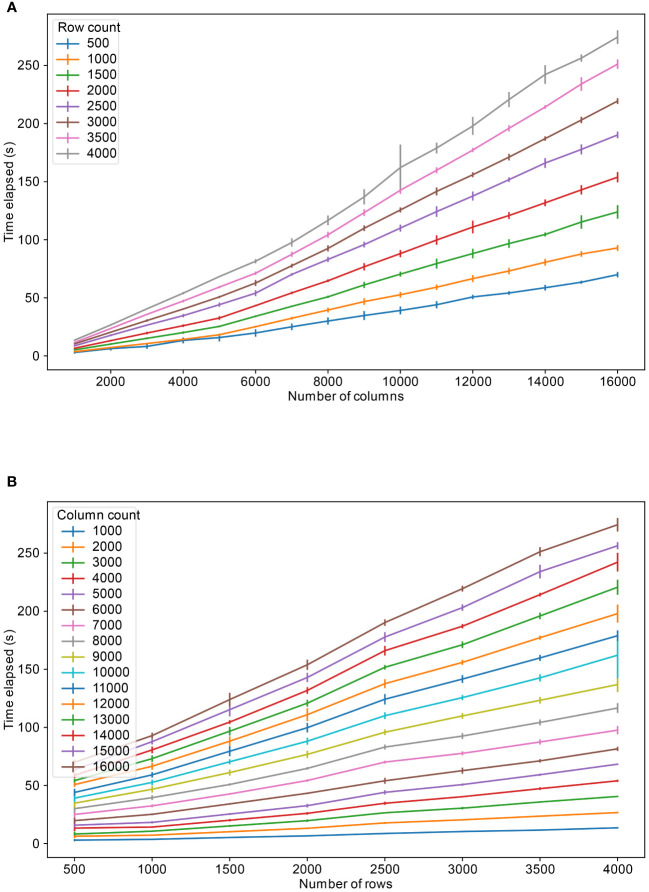
Table upload speeds for varying number of columns and rows, **(A)** for different fixed number of rows and **(B)** for different fixed number of columns. The colors refer to the fixed number of columns and rows respectively.

The database developed in this work was tested for its throughput capabilities. To carry out the tests, we generated CSV files containing values with various number of rows and columns. The synthetic data was generated to closely resemble the count matrices in microbiome data. The data upload results show that uploading a file to the database is mostly a linear relation with regards to the number of rows and columns (**Figure 3**).

As observed in **Figure 3B**, the time to upload a file roughly doubles with doubling of the column size, for total number of columns less than 10,000. However, for tables containing more than 10,000 columns, the upload time fluctuates depending on what the total size is. As stated above, in Supabase, there is maximum of 1664 columns per table. For practical purposes, for Postgres tables, such as in Supabase, a maximum of 2 (Huttenhower et al., 2023) (approximately 4 billion) rows could be stored in a table. Tables with more than 1664 columns were split into multiple sub-tables. These sub-tables can then be queried as single large table by establishing a common key, in our case sample donor id, for establishing the relation between the tables. For this reason, the column splitting limit per table was set to 500 for sub-table creation and leaving space for columns to be added later to the table. We did not observe significant upload time deviations based on rows and columns count combinations, for instance 3000 rows and 4000 columns table upload speed took almost equal time as 4000 rows and 3000 columns table upload, approximately 73 seconds.

**Figure 4:**
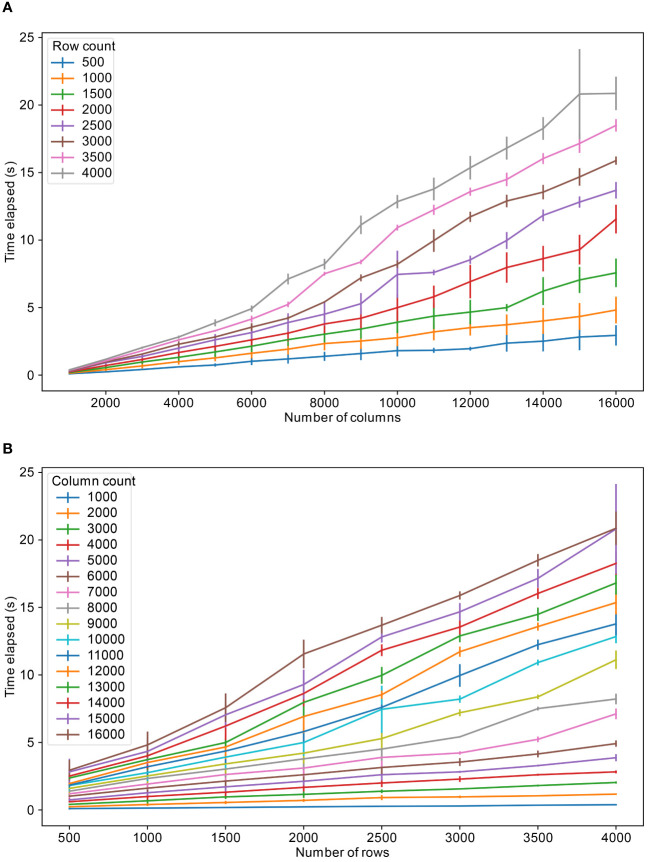
Table retrieval speeds for varying number of columns and rows, **(A)** for different fixed number of rows and **(B)** for different fixed number of columns. The colors refer to the fixed number of columns and rows respectively.

**Figures 4A, B** show the speed of retrieving data from the tables. To estimate this retrieval performance, a table and its related tables were looked up in the database and all rows corresponding to the tables were retrieved with an SQL query and later downloaded as csv file. The retrieval times show an approximately linear trend between the number of rows or columns queried and time for retrieval.

## 3 Discussions

In this article, we designed and built a FAIR-compliant database for storage of microbiome data. The FAIR principles were applied to the database platform itself as well as the data. Due to privacy-centric sensitivities associated with microbiome data, it is not possible to implement the FAIR principles in a strict manner, as that would require total transparency of data. As this database could include human data (DNA) and metadata, unfiltered publication of this data will both violate the privacy of the sample donors, and breach GDPR. The database was built using the open source Supabase platform. If authorized, users of the database can access, upload and download datasets. As shown in the results section, the linear scaling relation for data transfer speeds suggests that the database is capable of handling large volumes of data efficiently and proportionally to the underlying computing resources (computational power and storage). Two or more different data entries which were taken from the same donor by the same entity, can be either linked in relational datasets, or appended to one another. At the moment, due to Supabase functionality limits, appending data would first require the manual addition of the new columns to the existing table, before the data can be inserted. The main requirement for this functionality is for the providing entity to standardize its anonymization key (Abouelmehdi et al., 2018), thus giving each individual donor a unique identifier. This identifier can then be used across multiple sampling instances and multiple datasets, throughout time, to identify and group data entries per individual sample. The probability of a single sample having multiple measurements in our database is minimized. The only way this could be bypassed is if at a later date, the database would receive datasets from multiple uploading entities providing data from a single donor, then the donor would have had samples taken at more than one of those entities as duplicates. In that case, the donor would get a different unique identifier at each entity, leading to them not matching when all the data is combined in the database.

It can be concluded that the implementation of the FAIR principles in microbiome data research is possible to some extent, however, not completely. Other obstacles that we encountered were on the extent of the database implementation protocols as modules and the side effects that accompany it. Implementing these compliance principles can take a lot of time and thus can be expensive in terms of personnel hours. This comes hand in hand with technical expertise required to handle microbiome data, as the principles require all (meta)data to be standardized with Controlled Vocabulary Terms (CVT) from appropriate MIxS standards (Yilmaz et al., 2011) (minimum information about any (x) sequence), formatted and managed with regards to access and privacy. These obstacles have meant that the FAIR principles could not be fully implemented in their original essence, however, the remaining guidelines were still of great influence on the design of database. To overcome the above discussed constraints, we have provided three major modules in this work to adhere to the mandatory privacy principles and facilitate FAIR compliance.

### 3.1 FAIR data format compliance module

Microbiome shotgun data file formats, such as sequence files, FASTA or FASTQ, or count data, being plain-text format files, are not inherently FAIR compliant by themselves. It is therefore necessary to make them FAIR compliant by converting to universal Column Separated Values (CSV) format. The converter module converts such files into FAIR-complaint ‘.csv’ files containing appropriate data tables and a separate metadata ‘.txt’ files. During the data upload process, the database automatically scans the file types uploaded and raises appropriate error messages if the upload file is not strictly FAIR adherent. By creating comprehensive metadata about (or from) the dataset and strictly following the CSV format for data structure, both the metadata and data were made interoperable. This system allows the researchers to effortlessly input new data and revise current records in real-time and enables seamless querying, downloading, exhibition, and merging of the datasets with other standardized external sources.

### 3.2 Data privacy module

The data privacy module in our database functions in two distinct manners, (i) generation of privacy metrics and (ii) integration pipeline for federated learning. In cases of datasets containing social or demographic data of the microbiome data donors, the privacy module can be deployed to calculate privacy metrics, such as the entropy measure (to calculate the information predictability of data) or L-diversity score (Machanavajjhala et al., 2007). This way uploaded data that is personally identifiable can be easily identified. This can be later rectified by methods such as k-anonymity (Mayer et al., 2023) and ε- differential privacy (Dong et al., 2022). In our privacy module, we have provided an implementation of k-anonymity measure on the data. Additional functions can be easily added to this module at the data filtering step, if needed.

**Figure 5:**
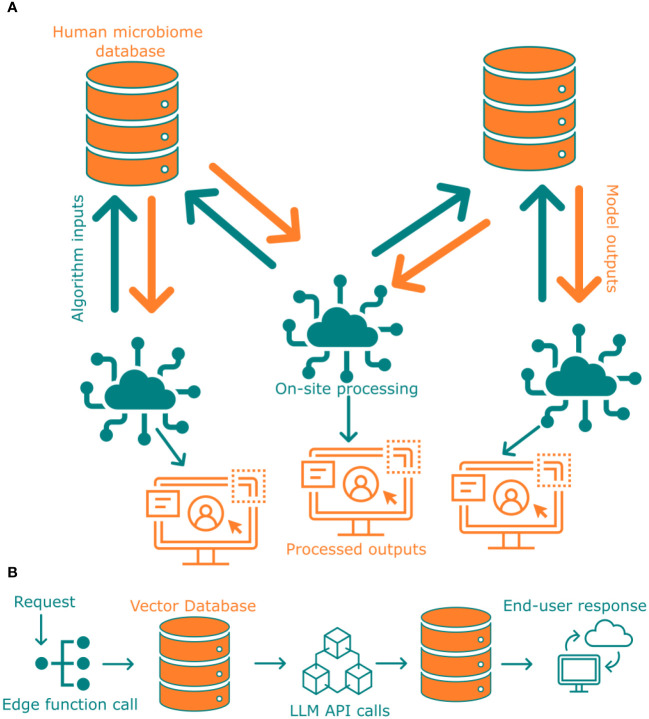
**(A)** Pipeline of federated learning from two different databases that are not connected with each other. **(B)** Deployment of Large Language Model with Supabase via edge functions.

There may arise situations where access to sensitive data may be necessary for completion of research objective. In such cases, federated learning could potentially mitigate the missing data situation. In federated learning, no sensitive data or metadata is shared with an end-user but rather all the computations required by the third-party or end user are carried out locally, this process is visually represented in **Figure 5A**. Only the results from this analysis will be shared with the end-user. A couple of steps are required to implement such a system to ensure that the results shared with the end-user are devoid of any unintended data leak. First, the output structure should be known beforehand, even before running any sensitive data analysis. Second, ensure that sensitive data cannot be reconstructed from the results. One way to pre-generate output against an end-user shared algorithm or program is to use synthetic microbiome data (Hittmeir et al., 2022). This enables an initial mock-run of the analysis to check against output structure and the results or graphs generated. Some outlier data points such as minority demographics could still be traceable in the generated results. To alleviate any further issues arising from identifiable data being present from the processed outputs, it is necessary to analyze the results before issuing them to the end-user. This can either be done by filtering the input with our privacy metric calculation module and/or by investigating only with algorithms compliant with privacy preserving analysis techniques (Gürsoy et al., 2022). To ensure data security, we provide a modular pipeline that enables the application of delegated learning techniques without revealing the confidential information to the user. Our system integrates this process directly into the database.

### 3.3 Accessibility enrichment module

Making scientific research findings accessible and understandable to the general public is a crucial component of FAIR data availability. Large language models can play a key role in facilitating this process by transforming complex scientific information into more relatable formats. These models can assist in creating educational materials, generating reports for policymakers, or answering questions from journalists and the media, ultimately improving knowledge dissemination on various levels within society. To ensure such accessibility at all levels of research, in this study, we have deployed a LLM (ChatGPT-based) via Supabase edge functions (Cao et al., 2020) to interpret data or findings (**Figure 5B**). Such an LLM, even if trained only against published literature from a study would enable broader knowledge dissemination to people of all strata, for instance could help answer a high school student’s question. This approach ensures FAIR is not restricted to scientific researcher, but could be widely used by non-researchers alike.

It is necessary to acknowledge the current lack of direct incentives to promote FAIR among researchers. Huttenhower et al. (2023) have provided some excellent pointers to incentivize FAIRification of data. Some include financial incentives such as prioritizing research proposals with FAIR compliance and penalizing others could promote FAIR culture among researchers. Even though they propose full deposition for publications, it may not be straight forward in countries where data privacy laws stifle FAIR principles. Workarounds such as the ones described in this manuscript should be considered to promote egalitarian scientific research irrespective of legal privacy considerations. Finally, the current study is limited to dealing with common datatypes and descriptors found in microbiome data. In presence of additional datatypes or descriptors, it may be necessary to implement additional privacy preservation metrics to ensure the data is non-identifiable. This is also true for validating outputs generated from Federated learning approaches. Here, the outputs could vary vastly based on the algorithms used. Even if the outputs themselves do not make the data identifiable, combining them could render it identifiable. Therefore, necessary algorithm screening techniques will need to be developed and implemented.

## 4 Conclusions

Our considerations and resulting procedures open opportunities for future thinking and research on conflicts between FAIR and privacy laws (GDPR). One concept that could use exploration, is the sharing of anonymization keys between healthcare data providers. This would mean that data from two different entities on the same patient could be merged, possibly creating new connections, trends and scientific findings. Naturally, this also brings along new concerns for privacy and data security, however, seeing as patient data is already being shared between the world’s healthcare providers, it could be a logical step to share patient’s anonymized aliases as well. A second field of interest is how the research in this manuscript relates to the rest of the world. In Europe, on which this work is based, GDPR strictly govern data privacy and data use. In other parts of the world, rules and regulations are different, meaning that if this research had been conducted somewhere else, it could have led to different procedures. The need for a harmonized global view on data sharing in healthcare research is underscored by the upcoming efforts in this field in Asia and Africa. Sharing healthcare data beyond national and continental borders will greatly advance global healthcare research, especially in the new territory of microbiome and genomic data research. An exploration of these global cooperative efforts, including the digital and legal infrastructure needed to support them, could be a catalyst for new scientific advancements and findings. In future, collaborations between healthcare professionals, computational modelers, data scientists and AI experts can be crucial in maximizing the potential of data and technology in healthcare. Therefore, it is important to promote interdisciplinary education and training programs that bring together healthcare professionals, data scientists, and AI experts. The user friendly nature of the framework developed in this manuscript should help overcome technical difficulties associated with using FAIR microbiome data. However, it is still crucial to ensure that future physicians are better prepared to leverage these tools in delivering patient care.

## Data availability statement

The original contributions presented in the study are included in the article/supplementary material. Further inquiries can be directed to the corresponding author.

## Author contributions

MD: Investigation, Methodology, Software, Visualization, Writing – original draft, Writing – review & editing. NZ: Data curation, Investigation, Methodology, Software, Visualization, Writing – original draft, Writing – review & editing. RW: Writing – original draft, Writing – review & editing. DM: Writing – original draft, Writing – review & editing. BB: Methodology, Writing – original draft, Writing – review & editing. EZ: Writing – original draft, Writing – review & editing. AH: Writing – original draft, Writing – review & editing. VS: Conceptualization, Data curation, Investigation, Methodology, Software, Supervision, Visualization, Writing – original draft, Writing – review & editing.
